# Antihepatitis B Surface Antigen and Hepatitis C Antibodies among Pregnant Women in an Urban Area of Mwanza City, Tanzania

**DOI:** 10.1155/2019/7917894

**Published:** 2019-06-18

**Authors:** Elieza Chibwe, Vitus Silago, Edwin Kajoro, Muhsin Juma, Emmanuel Mkumbo, Caroline A. Minja, Fridolin Mujuni, Stephen E. Mshana, Mariam M. Mirambo

**Affiliations:** ^1^Department of Obstetrics and Gynecology, Weill Bugando School of Medicine, Catholic University of Health and Allied Sciences, P.O. Box 1464, Mwanza, Tanzania; ^2^Department of Microbiology and Immunology, Weill Bugando School of Medicine, Catholic University of Health and Allied Sciences, P.O. Box 1464, Mwanza, Tanzania; ^3^Department of Biochemistry and Molecular Biology, Weill Bugando School of Medicine, Catholic University of Health and Allied Sciences, P.O. Box 1464, Mwanza, Tanzania

## Abstract

**Background:**

Hepatitis B and hepatitis C viruses (HBV and HCV) are life-threatening infections of public health importance due to their association with cirrhosis and hepatocellular carcinoma. Despite HBV being moderately endemic in many low-income countries, there is no routine HBV vaccination among child bearing aged women making them at risk of transmitting infections to the foetus during pregnancy. This study investigated the seroprevalence of antihepatitis B surface antibodies (anti-HBs) and HCV antibodies among pregnant women in Mwanza city to provide data that can be used in devising preventive strategies.

**Methods:**

A cross-sectional hospital-based study involving 339 pregnant women was conducted between June and July 2017. Data were collected using structured data collection tool. Detection of anti-HBs was performed using enzyme immunoassay while qualitative rapid immunochromatographic tests were employed to detect HCV antibodies. Data was analyzed by using STATA version 13.

**Results:**

The mean age of the study participants was 25.6±5.8 years. The prevalence of anti-HBs was 85/339 (25.1%, 95% CI: 20.4-29.6) while that of HCV antibodies was 1/333 (0.3%, 95% CI: 0.1-0.4). By univariate logistic regression analysis, increase in age (OR: 1.04, 95% CI: 1.00-1.09, P=0.03), unknown HIV status (OR: 0.3, 95% CI: 0.11-0.79, P=0.035), and multigravidity (OR: 2.12, 95% CI: 1.18-3.8, P=0.038) were significantly associated with anti-HBs seropositivity.

**Conclusion:**

A significant proportion of pregnant women have anti-HBs while the seroprevalence of HCV is low among pregnant women in the city of Mwanza. Routine screening of HBV among pregnant women coupled with appropriate management should be emphasized in developing countries. Further studies to determine seroprevalence of HCV are recommended across the country.

## 1. Introduction

Hepatitis B virus (HBV) and hepatitis C virus are major public health concerns particularly in the sub-Saharan Africa and Asian countries. HBV causes over one million deaths and infections to over 350 million people worldwide. Most of individuals in endemic areas have been infected vertically or during childhood [1]. Similarly, HCV infection is common in many countries worldwide [2]. The World Health Organization (WHO) estimates that 3% of the world's populations are chronically infected with HCV with high prevalence reported in the sub-Saharan Africa [3, 4]. Furthermore, hepatitis A and E have been documented to have adverse outcomes in pregnancy [5, 6]. Despite percutaneous inoculation being the well-known mode of transmission of HCV, other previous reports documented other transmission routes such as unprotected sexual intercourse, occupational accidents, and vertical transmission [7].

Infections with HBV and HCV during pregnancy have been associated with high risk of vertical transmission which may result in neonatal hepatitis. The highest risk (80%-90%) of chronic infection has been observed among neonates with neonatal hepatitis [8, 9]. Other complications include low birth weight and premature delivery during the acute phase as well as antepartum hemorrhage and preterm delivery during chronic phase [10]. Understanding epidemiology of viral hepatitis during pregnancy is important as the information can be used in devising appropriate control measures.

Different studies in Africa have documented the magnitude of viral hepatitis during pregnancy [11–14] using HBsAg with very few studies assessing other markers such as anti-HBs. Despite its importance there is scarcity of data in some of African countries including Tanzania on the magnitude of this infection. In Tanzania, HBV vaccine has been introduced in childhood immunization programme about 5 years ago leaving out the adult population including women of reproductive age. This puts these women at high risk of transmitting the infection during pregnancy. This study was conducted to investigate the prevalence and factors associated with anti-HBs (natural immunity) and HCV antibodies among pregnant who had no history of HBV vaccination and who were HBsAg negative, the information that may be useful in accelerating efforts to consider incorporating HBV vaccine in other populations including reproductive aged women in endemic areas across the globe.

## 2. Materials and Methods

### 2.1. Study Design and Study Area

This was a cross-sectional hospital-based study which was conducted from June to July 2017 at Makongoro antenatal clinic in Mwanza city. Mwanza is the second largest city in Tanzania; it is located in the southern shores of Lake Victoria. Makongoro Health Centre is primarily a reproductive and child healthcare facility found in Ilemela district. The clinic serves both Nyamagana and Ilemela districts, with estimated attendance of 60-70 pregnant women per working day.

### 2.2. Study Population, Inclusion and Exclusion Criteria

The study included all consented pregnant women aged 18 and above at different gestation ages attending Makongoro antenatal clinic during the study period while all women with unknown gestation ages were excluded. The study ecluded all women with history of HBV vaccination and those who were HBsAg positive.

### 2.3. Sample Size Estimation and Sampling Technique

Sample size was estimated by using Kish Leslie formula (1965) [15] using the prevalence of 30.2% [16]. The minimum sample size obtained was 324 pregnant women. However, a total of 339 women were enrolled. Serial sampling based on inclusion and exclusion criteria was used to recruit study participants until the desired sample size was reached.

### 2.4. Data Collection, Sample Collection, and Laboratory Procedures

Sociodemographic and other relevant information was collected by using pretested structured data collection tool. Venous blood was collected from all consented participants. Blood was aseptically drawn from the cephalic vein using sterile disposable 5 ml syringes and placed in labeled plain vacutainer tubes (Becton, Dickinson and Company, USA). The tubes were kept at upright position at 21-25°C and transported to the CUHAS multipurpose laboratory for processing. The sera were separated, placed in labeled cryovials, and stored at -80°C until processing.

Detection of anti-HBs was done using commercial Sandwich Enzyme Immunoassay (EIA) according to the manufacturer's instructions (SIEMENS-Enzygnost Anti-HBs II, Marburg, Germany) while HCV antibodies were detected by using immunochromatographic test as per manufacturer's instructions, ACON one-step HCV test strip (ACON Laboratories, Inc., CA 92121, USA; DIALAB GmbH, Austria).

### 2.5. Data Management and Analysis

Data were entered into Excel sheet, cleaned, coded, then transferred into STATA version 13.0 for analysis. Proportions were used to summarize categorical variables while mean (standard deviation) and median (interquartile range) were used for continuous variables. Ranksum Mann-Whitney test was used to compare medians. A stepwise logistic regression model was used to test association between outcome variables and associated factors whereby the variables with P value of <0.2 on univariate analysis were subjected to multivariate logistic regression analysis. Odds ratio (OR), P values, and 95% confidence interval (CI) were noted. A P value of <0.05 was considered as statistically significant.

## 3. Results

### 3.1. Sociodemographic Characteristics of the Study Participants

The mean age of the study participants was 25.6±5.8 years while the median gestation age was 23 (IQR: 14-28) weeks. The majority of the participants, 309 (91.5%), were married. Most of the participants, 218 (64.3%), had either no formal education or primary education while about half, 186 (54.9%), of the participants were multigravida (Table 1).

### 3.2. Prevalence of Anti-HBs and HCV Antibodies and Associated Factors among Pregnant Women in Mwanza City

The seroprevalence of anti-HBs was found to be 85 (25.07%, 95% CI: 20.4-29.6). The mean age of anti-HBs seropositive women was significantly higher than that of their counterparts (26.8±5.8 vs. 25.2±5.7, P=0.03). On univariate logistic regression analysis, increase in age 26.8±5.8 (OR: 1.04, 95% CI: 1.00-1.09, P=0.03), unknown HIV status (OR: 0.3, 95% CI: 0.11-0.79, P=0.035), and multigravidity (OR: 2.12, 95% CI: 1.18-3.8, P=0.038) were significantly associated with anti-HBs seropositivity. By multivariate logistic regression analysis, only unknown HIV status (OR: 0.32, 95% CI: 0.12-0.86, P=0.02) was found to be associated with anti-HBs seropositivity (Table 2). Among 333 women who were tested for HCV antibodies, only one (0.03%, 95% CI: 0.1-0.4) was found to be seropositive. None of the factors was found to predict HCV seropositivity among pregnant women in Mwanza city.

## 4. Discussion

This is the first study to assess the level of anti-HBs and HCV antibodies among pregnant women population in Mwanza city. The seroprevalence of anti-HBs in the current study was found to be high, which is comparable to a previous study in Kenya that reported anti-HBs prevalence of 30.2%[16]. Nonetheless, the seroprevalence in this study is significantly higher than what has been documented in Brazil and Bangladesh [17, 18] with prevalence of 5.7% and 8.5%, respectively. On the contrary, the seroprevalence in this study is lower than that of a previous study in China which reported seroprevalence of 58.55% [19]. The possible explanation for such discrepancies could be geographical variation, endemicity status as per WHO [20], and exposure to the risk factors. Tanzania is among the countries under moderate endemicity which might be different from other countries like China where the endemicity is high moderate and Brazil where the endemicity is low (http://www.hpsc.ie/a-z/EMIToolkit/appendices/app22.pdf).

In this study, only unknown HIV status was found to predict anti-HBs seropositivity. This observation is different from the previous studies which did not show any association between anti-HBs and HIV status[16, 19]. This observation can be explained by the fact that the HIV status reported in this study was based on antenatal records which might not be reliable to some of the participants. There is a possibility that some of the participants who claimed to have unknown status have positive results which might explain such association. Further studies to confirm this association are recommended in this setting.

Another factor which showed association with anti-HBs seropositivity was increased age which is similar to the previous study done in Bangladesh [18]. A possible explanation for such association could be the fact that aged women are more likely to be exposed to the risk factors compared to young women.

Regarding HCV seropositivity, the seroprevalence was found to be 0.03% which is comparable to other studies done in Sudan and Nigeria that reported prevalence of 0.6% and 0.4%, respectively [21]. In comparison to other studies done in Egypt, Ivory Coast, and Rwanda which reported prevalence of 6.1%, 3.6%, and 2.4% [21, 22], the reported HCV seroprevalence in the current is significantly low. The possible explanation could be different geographical distribution and exposure to risk factors in relation to the study population [21–25]. Another possible explanation could be differences in sensitivity and specificity of the test used in different studies. In the current study, the test used has sensitivity and specificity of 98.9% and 99.2% which might be different from other previous studies.

As a limitation, in the present study anti-HBc antibodies were not tested. However, the population studied had not received HBV vaccination and were HBsAg negative, so the results obtained are highly indicative of natural anti-HBs antibodies.

## 5. Conclusions

A substantial number of pregnant women have natural anti-HBs while the prevalence of HCV antibodies is low among pregnant women in the city of Mwanza. However, a significant proportion of these women are susceptible to HBV infection necessitating the need to ensure all reproductive aged women are protected against HBV. Routine screening of HBV infection among pregnant women coupled with appropriate management should be emphasized in resource limited countries where the viral hepatitis is endemic. Further studies on HCV in different cities across the country are highly recommended.

## Figures and Tables

**Table 1:** Sociodemographic and clinical characteristics of 339 pregnant women attending Makongoro antenatal clinic.

Participant characteristics	Number	Percent/Mean/Median
*Age*	339	25.6±5.8
Gestation	339	23 (IQR:14-28)
Marital status		
Married	309	91.5
Unmarried	30	8.5
HIV status		
Negative	281	82.8
Positive	10	2.9
Unknown	48	14.2
Education level		
None/primary	218	64.3
Secondary	95	28.2
Tertiary	26	7.7
Gravidity		
Prime gravida	115	34.0
Gravida 2,3,4	186	54.9
Gravida >5	38	11.2
Anti-HBs Negative	254	74.93%
Anti-HBs Positive	85	25.07%
*Titres*		
10-100IU/ml	63	18.58%
100-200IU/ml	10	2.95%
>200IU/ml	12	3.54%

**Table 2:** Factors associated with anti-HBs seropositivity among 339 pregnant women attending Makongoro antenatal clinic.

			Univariate analysis		Multivariate analysis	
Variable	Immuno (Negative)	Immuno (Positive)	OR[95%CI]	P-value	OR[95%CI]	P-value
	Median%(IQR)	Median%(IQR)				
*Age*	25.2±5.7	26.8±5.8	1.04[1.00-1.09]	0.033	1.02[0.97-1.08]	0.358
*Gestation *	21.5(IQR:14-28)	24(IQR:16-28)				
*Marital S.*						
Single	23(76.67)	7(23.33)	1			
Married	231(74.76)	78(25.24)	1.1[0.45-2.68]	0.818		
*HIV*						
Negative	203(72.24)	78(27.76)	1			
Positive	8(80)	2(20)	0.65[0.13-3.13]	0.592	0.47[0.92-2.39]	0.36
Unknown	43(89.58)	5(10.42)	0.3[0.11-0.79]	0.035	0.32[0.12-0.86]	**0.02**
*Education*						
None/Pri.	159(72.94)	59(27.06)	1			
Second.	76(80)	19(20)	0.67[0.37-1.2]	0.405		
Tertiary	19(73.08)	7(26.92)	0.99[0.39-2.48]	0.988		
*Blood splash*						
No	241(75.79)	77(24.21)	1			
Yes	13(61.90)	8(38.10)	1.92[0.76-4.8]	0.155	1.79[0.68-4.67]	0.23
*Gravidity*						
Prim gravid	96(83.48)	19(16.52)	1			
Gravid 2,3,4	131(70.43)	55(29.57)	2.12[1.18-3.8]	0.038	1.75[0.9-3.42]	0.36
Gravid 5 ->	27(71.05)	11(28.95)	2.05[0.87-4.84]	0.099	1.32[0.44-3.91]	0.61

## Data Availability

All data are included in the manuscript. Raw data is available upon request, and the request should be made to the Director of Research and Innovation, Catholic University of Health and Allied Sciences.
